# Supplementary material for: Is the Canadian Healthy Eating Index 2007 an Appropriate Diet Indicator of Metabolic Health? Insights from Dietary Pattern Analysis in the PREDISE Study
Source: Nutrients. 2019 Jul 14;11(7):1597. doi: 10.3390/nu11071597 (PMC6683076; doi:10.3390/nu11071597)
Supplement: Supplementary file 1 [file nutrients-11-01597-s001.pdf]

Supplemental material

Supplemental Table S1: Description of the Canadian Healthy Eating Index 2007 (C-HEI 2007) scoring

Component	Range of scores[1]	Scoring criteria
**Adequacy[2]**	**0 to 60 points**	
Total vegetables and fruit	0 to 10 points	Minimum: 0<br>Maximum: 7 to 8 servings*
Whole fruit	0 to 5 points	Minimum: 0<br>Maximum: 1.5 to 1.7 servings (21% of recommendations for total vegetables and fruit)*
Dark green and orange vegetables	0 to 5 points	Minimum: 0<br>Maximum: 1.5 to 1.7 servings (21% of recommendations for total vegetables and fruit)*
Total grain products	0 to 5 points	Minimum: 0<br>Maximum: 6 to 8 servings*
Whole grains	0 to 5 points	Minimum: 0<br>Maximum: 3 to 4 servings (50% of recommendations for total grain products)*
Milk and alternatives	0 to 10 points	Minimum: 0<br>Maximum: 2 to 3 servings*
Meat and alternatives	0 to 10 points	Minimum: 0<br>Maximum: 2 to 3 servings (150 to 225 grams)*
Unsaturated fats	0 to 10 points	Minimum: 0<br>Maximum: 30 to 45 grams
**Moderation[3]**	**0 to 40 points**	
Saturated fats	8 to 10 points	Minimum 7% to 10% of total energy intake
	0 to 8 points	10% to maximum 15% of total energy intake
Sodium	8 to 10 points	Adequate intake (1500 mg/day) to tolerable upper intake level (2300 mg)/day)
	0 to 8 points	Tolerable upper intake (2300 mg/day) level to twice tolerable upper intake level (4600 mg/day).
"Other foods"	0 to 20 points	Minimum: 5% or less of the total energy intake<br>Maximum: 40% or more of the total energy intake

Adapted from: Garriguet (2009)

Total score /100

*Minimum or maximum number of servings are sex and age specific and determined per day by the 2007 Eating well with the Canada's Food Guide (Government of Canada, H.C. Eating Well with Canada's Food Guide: A Resource for Educators and Communicators [Health Canada, 2007] Available online: http://www.hc-sc.gc.ca/fn-an/pubs/res-educat/res-educat-eng.php (accessed on Oct 14, 2016).)

1. Range of scores used for adults.

2. For adequacy components, 0 points for minimum, 5 or 10 points for maximum or more, and proportional for amounts between minimum and maximum.

3. For moderation components, 10 or 20 points for minimum or less, 0 points for maximum or more, and proportional for amounts between minimum and maximum.

Supplemental Table S2: Description of the Alternative Healthy Eating Index 2010 (AHEI) scoring

**Components**	**Criteria for minimum score (0)**	**Criteria for maximum score (10)**
Vegetables, servings/ day	0	≥5
Fruit, servings/day	0	≥4
Whole grains, g/day	0	
Women		75 (approximately 5 servings/day)
Men		90 (approximately 6 servings/day)
Sugar-sweetened beverages and fruit juice, servings/day	≥1	0
Nuts and legumes, servings/day	0	≥1
Red/processed meat, servings/day	≥1.5	0
Trans fat, % energy	≥4	≤0.5
Long chain (n-3) fats (EPA+DHA), mg/day	0	250
PUFA, % energy	≤2	≥10
Sodium, mg/day	Highest decile	Lowest decile
Alcohol, drinks/day[1]		
Women	≥2.5	0.5-1.5
Men	≥3.5	0.5-2.0
Total	0	110

1. In the design of the AHEI, authors assigned the highest score to moderate, and the lowest score to heavy, alcohol consumers. The nondrinkers received a score of 2.5. One drink is 4 oz of wine, 12 oz of beer, or 1.5 oz of liquor (1 oz = 28.35 g).
Adapted from: Chiuve et al. (2012)

Total score /110

Supplemental Table S3: Food groups used for the reduced rank regression analysis

Food groups	Description*
Dark Green Vegetables	All dark green vegetables. One portion is equivalent to 125 ml except for leafy vegetables for which the portion size is 250 ml.
Orange Vegetables	All orange vegetables (e.g. carrots, pumpkin, squash, sweet potatoes and yam). Includes some orange fruits (apricot, cantaloupe, mango, nectarine, papaya and peach). One portion is equivalent to 125 ml except for some fruit (3 apricots, ½ papaya, 1 medium nectarine or peach).
Other Vegetables	Vegetables that are not included in Dark Green and Orange Vegetables. One portion is equivalent to 125 ml except for leafy vegetables for which the portion size is 250 ml.
Whole Fruit	All fruit except orange fruit included in the Orange Vegetables. Fruit juices are excluded. One portion defined as 125 ml or the equivalent.
Fruit Juice	All 100% fruit juices. One portion defined as 125 ml.
Whole Grains	Grain product for which the first ingredient is a whole grain product. One portion defined as 125 ml of cooked grain or the equivalent of 30 g of processed grain.
Refined Grains	All grain products except those included in the Whole grains group. One portion defined as 125 ml of cooked grains or the equivalent of 30 g of processed grain.
Yogurt	All yogurt and kefir. One portion defined as 175 g except for yogurt drink for which the portion is 200 ml.
Milk	All types of milk excluding fortified soy beverages. One portion defined as 250 ml.
Other Dairy Products and Alternatives	All other milk and alternative excepted milk, yogurt and kefir. Includes fortified soy beverages. One portion defined as 250 ml for milk and 50 g for cheese.
Nuts and Legumes	Includes all types of nuts, legumes and soy product beside fortified soy milk. One portion is defined as 35 g for nuts and nut butter, 120 g for legumes, 150 g for soy products.
Fish and Seafood	All types of fish and seafood. One portion defined as 75 g.
Poultry	All types of poultry. One portion defined as 75 g.
Eggs	One portion defined as two eggs or 100 g.
Red and Processed Meat	Includes beef, pork, veal, lamb, game meat, horse and giblets. Processed meat includes cold cuts and sausages. One portion defined as 75 g.
Sugar-Sweetened Beverages	Portions defined as 227 g (8 oz of 28.35 g). Included fruit-flavoured drinks, soft drinks, sports drinks, energy drinks, sugar-sweetened coffee and tea. Excludes 100% fruit juices and flavoured milks.
Other Foods	Includes food items with very high content in fat, sugar and/or salt and not included in Canadian food guide groups. Excludes sugar-sweetened beverages. As there is no defined portion for most of the food in this group, one portion is defined as 1 kcal.

* Portions are measured in volume according to the Canadian Food Guide guidelines. Conversion from volume to weight with the Canadian Nutrient File.

Supplemental Table S4: Response variables used to derive the dietary patterns across quintiles (Q) of the selected dietary pattern (DP) (N=998).

	Quintiles of DP										P for trend
	Q1		Q2		Q3		Q4		Q5		
	**Means**	SD	**Means**	SD	**Means**	SD	**Means**	SD	**Means**	SD	
HDL-cholesterol (mmol/L)	**1.5**	0.5	**1.5**	0.5	**1.4**	0.4	**1.4**	0.4	**1.3**	0.4	<0.001
Triglycerides (mmol/L)	**1.1**	0.6	**1.3**	0.8	**1.4**	0.7	**1.4**	1.0	**1.6**	1.4	<0.001
HOMA-IR	**3.5**	2.7	**3.8**	3.2	**4.1**	4.3	**3.8**	2.5	**4.4**	3.2	0.06
Waist circumference (cm)	**85.5**	14.6	**88.7**	13.5	**93.0**	17.3	**93.1**	15.5	**99.7**	16.9	<0.001
Mean blood pressure (mmHg)	**85.2**	11.0	**86.9**	10.3	**88.8**	10.9	**89.3**	10.5	**91.1**	10.5	<0.001

Supplemental Table S5: Prevalence ratio of the metabolic syndrome components across quintiles of adherence (5th vs 1st quintiles) of Healthy Eating Index (C-HEI 2007), Alternate Heathy Eating Index 2010 (AHEI), the selected dietary pattern (DP) and the Modified Healthy Eating Index 2007 (Modified C-HEI) (N=998).

		**Prevalence ratio** (95% CI)[1]			
	% of cases	C-HEI 2007	AHEI	DP	Modified C-HEI
**Blood pressure**	19.0	**0.58***	**0.42*****	**0.50****	**0.42****
(≥130/85)		(0.37, 0.92)	(0.27, 0.67)	(0.33, 0.78)	(0.26, 0.68)
**Fasting blood glucose**	20.3	**0.72**	**0.90**	**0.69**	**0.65***
(≥5.6 mmol/L)		(0.50, 1.01)	(0.59, 1.36)	(0.45, 1.06)	(0.44, 0.97)
**Triglycerides**	29.9	**0.60*****	**0.50*****	**0.44*****	**0.46*****
(≥1.7 mmol/L)		(0.45, 0.80)	(0.36, 0.70)	(0.31, 0.62)	(0.34, 0.63)
**HDL-cholesterol**	25.5	**1.00**	**0.50****	**0.90**	**0.75**
(women: <1.3 mmol/L; men: <1.0 mmol/L)		(0.72, 1.39)	(0.34, 0.74)	(0.61, 1.32)	(0.53, 1.06)
**Waist circumference**	37.9	**0.78**	**0.48*****	**0.55*****	**0.60*****
(women: ≥88 cm; men: ≥102 cm)		(0.60, 1.01)	(0.36, 0.64)	(0.42, 0.73)	(0.45, 0.78)

Binomial regression adjusted for age (continuous), physical activity (continuous), energy (continuous), smoking status (2 categories), sex (2 categories), ethnicity (2 categories), marital status (2 categories) and education (3 categories)

P for linear trend across prevalence ratio* P<0.05, **P<0.01, ***P<0.001

1. Reference is the 5th quintile (strongest adherence and lowest diet quality) for DP and the 1st quintile (lowest adherence and lowest diet quality) for C-HEI 2007, AHEI and Modified C-HEI.
